# Recurrent Synovial Sarcoma with Breast and Pulmonary Nodule: A Case Report

**DOI:** 10.31729/jnma.8408

**Published:** 2024-01-31

**Authors:** Prajwal Khanal, Biraj Baral, Prasamsa Pande, Sohil Neupane, Rinku Joshi

**Affiliations:** 1 Kathmandu Medical College and Teaching Hospital, Sinamangal, Kathmandu, Nepal; 2 Department of Medicine, Shree Birendra Hospital, Cnhauni, Kathmandu, Nepal

**Keywords:** *case reports*, *chemotherapy*, *immunohistochemistry*, *synovial sarcoma*

## Abstract

Synovial sarcoma is a mesenchymal tumour with partial epithelial differentiation. About 85-90% of SS occur in the extremities. We present a case of a 44-year-old woman diagnosed with recurrent synovial sarcoma with breast and pulmonary nodules. The primary treatment for synovial sarcoma is wide surgical excision, while chemotherapy is reserved for metastatic cases. In the first-line metastatic setting, combination treatment with adriamycin and ifosfamide is administered. Despite the unfavourable prognosis, the patient's extended survival is fortunately not the typical outcome.

## INTRODUCTION

Synovial sarcoma (SS) is a mesenchymal tumour with partial epithelial differentiation that presents mainly in adolescents and adults younger than 30 years of age.^1,2^ It is a relatively rare tumour, with an estimated diagnosis rate of 1-2 people per million population.^2^ The tumour is not limited to the synovium and can originate anywhere in the body. It earned its name because of its anatomical location and histological resemblance to normal synovial tissue and is often found in the arm, leg, or foot, and near joints such as the wrist or ankle can also form in soft tissue such as the lung.^3^ We present a case of a 44-year-old female, diagnosed with recurrent synovial sarcoma with breast and pulmonary nodules.

## CASE REPORT

A 44-year-old patient presented with a chief complaint of a mass over the medial aspect of the left thigh that had been gradually increasing over the past 13 years. The patient had previously undergone four surgical procedures. The first surgical procedure for incision and drainage was carried out due to a suspected hematoma of unknown origin. Suspicions of recurrent hematoma led to a second surgical excision being performed two years later. At the age of 38 years, she was initially diagnosed with synovial sarcoma in the left thigh, which was confirmed by immunohistochemistry studies. Tumour cells were immunoreactive for transducer-like enhancer of split 1 (TLE1) and cluster of differentiation 99 (CD99) with a score of 4+ and 3+, susceptible and specific for monophasic synovial sarcoma diagnosis. The immunoreactivity of neoplastic cells to CD31, CD34, CK, desmin, and S-100 was absent. The third wide local surgical excision was performed 5 years earlier for synovial sarcoma.

**Figure 1:**
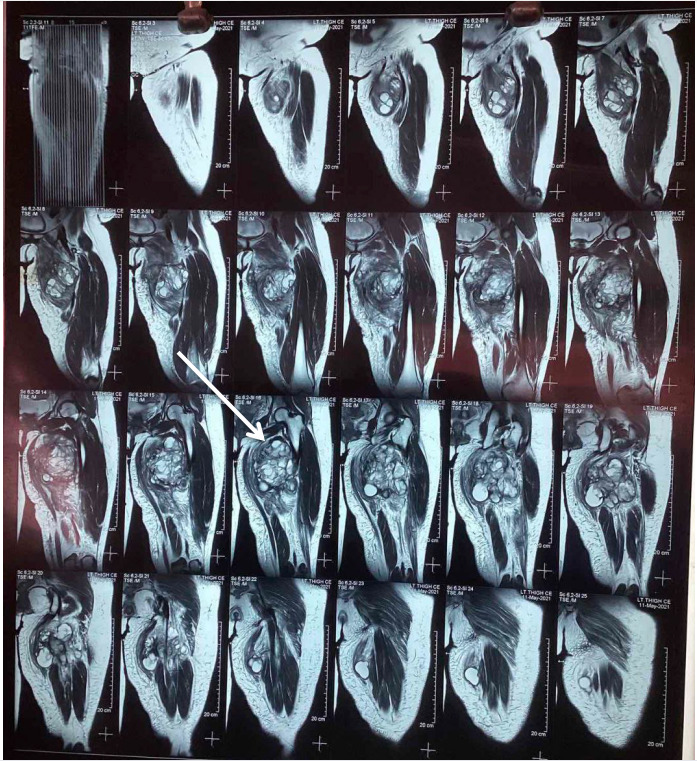
T2 Sequenced MRI showing Triple sign positive.

A fluorodeoxyglucose (FDG)-avid mass in the medial thigh musculature was discovered during a positron emission tomography-computed tomography (PET- CT) scan, which suggests that there is a residual lesion of synovial sarcoma. Metastatic suspicion arose as a result of the presence of FDG-avid nodules in the right lung, as well as an FDG-avid nodule observed in the upper inner quadrant of the left breast. There was no evidence of distant skeletal metastasis as suggested by the 3 Technetium methyl diphosphonate (3Tc-MDP) whole-body bone scan. Tracer uptake was only noted in the soft tissue of the left thigh. Then the patient was started on pre-operative radiotherapy for 30 days. A magnetic resonance imaging (MRI) of the left thigh showed a large heterogeneously enhancing mass 103 x 122 x 130 mm involving the medial compartment, with T1 low and T2/fat-saturated high signal intensity. The image below shows a T2-sequenced MRI showing a demarcated multilobulated mass (Figure 1).

The three signals appear positive, which indicates the presence of areas that exhibit three different signal intensities: areas of necrosis exhibit very high signal, soft tissue components exhibit high signal, and dystrophic calcifications exhibit low signal intensity. The fat plane is seen preserved with no bone involvement or invasion. Two years before the current time, the fourth surgical excision was performed to address the synovial sarcoma in the left thigh.

A contrast-enhanced computed tomography (CECT) scan of the chest, abdomen, and pelvis showed multiple enhancing nodules in the right lung. The largest nodules were found in the right middle lobe. A small nodule was also found in the superior segment of the left lower lobe. Additionally, a small enhancing nodule was detected in the upper inner quadrant of the left breast. An ill-defined enhancing lesion was noted in the medial compartment of the left thigh. These findings were consistent with recurrent synovial sarcoma.

Chemotherapy was initiated as part of the treatment plan. The initial regimen consisted of ifosfamide and adriamycin chemotherapy. The patient was scheduled to undergo six cycles of chemotherapy, receiving ifosfamide (2 gm/m^2^) for 5 days and adriamycin (25 mg/m^2^) for 3 days. In the second cycle, the treatment plan was adjusted, and cisplatin was added to the regimen. Subsequent chemotherapy sessions followed the same protocol of ifosfamide, adriamycin, and cisplatin regimen.

During the follow-up MRI, a recurrence of synovial sarcoma was again observed, with a large heterogeneously enhancing mass in the medial compartment of the left thigh. Pulmonary nodules had decreased in size compared to previous imaging, while the left breast nodule remained stable. The CECT scan revealed an increase in the size of bilateral lung nodules, with no changes in the left breast nodule and a decrease in the size of the ill-defined lesion in the left thigh.

## DISCUSSION

Synovial sarcoma comprises 5-10% of all soft tissue sarcoma.^1^ Synovial sarcoma is a relatively rare tumour with an incidence rate of 0.177 per 100,000 and a prevalence rate of 0.65 per 100,000.^2^ About 85-90% of SS occur in the extremities.^3^ SS is primarily encountered in adolescents or young adults at 15-40 years of age. Males are affected more often than females. Synovial sarcoma is an aggressive malignancy with a poor prognosis. The expected 5-year survival is between 50-60% in adults, and 5-year metastasis-free survival is between 40 to 60%.^2^ Younger age, smaller tumour size, extremity location, heavy calcification, negative surgical margins, and lower stage are favourable prognostic factors.^4^ Perioperative radiotherapy and the absence of osseous or neurovascular invasion are also associated with a better prognosis.^4^ The different types of synovial sarcoma are classified based on their microscopic appearance which includes- monophasic fibrous synovial sarcoma, biphasic synovial sarcoma, monophasic epithelial synovial sarcoma, calcifying synovial sarcoma and poorly differentiated synovial sarcoma.^5^ The monophasic fibrous type is the commonest while the monophasic epithelial subtype is of the rarest variety.

SS is characterized by a specific chromosomal translocation between the SS18 gene on chromosome 18 and one of the several synovial sarcoma X genes on chromosome X.^6,7^ The majority of synovial sarcomas are slow-growing and the mean duration of symptoms before diagnosis is approximately 2 years.^8^ Similarly, in our case, the patient experienced painless swelling for approximately six years before the diagnosis of synovial sarcoma. An immunohistochemistry panel is to be done for proper diagnosis of the case. TLE1 is an excellent marker in distinguishing SS from other soft- tissue malignancies. Tumor cells were immunoreactive with a 4+ grade for the TLE1 marker in our case, along with a 3+ grade for CD99 establishing a proper diagnosis for SS. Our case is a typical case of monophasic fibrous Synovial sarcoma which was diagnosed 6 years back with immunohistochemistry methods and consistent MRI findings. A negative margin surgical excision was performed. SS is associated with local recurrence and distant metastasis. Like other soft tissue sarcomas, MRI with and without contrast is the gold standard for synovial sarcoma imaging. The presence of the Triple sign, as observed in our case, can be considered a characteristic finding.^9^ Surgical excision is the treatment of choice, and the recurrence rate ranges from 28% to 36% even with adequate surgical and adjunctive therapies.^3^ She was diagnosed with recurrent synovial sarcoma with metastasis to the left breast and middle lobe of the right lung using a PET scan. As suggested by different works of literature, in the first-line metastatic setting, combination treatment with adriamycin and ifosfamide is a preferred option in fit patients.^10^ Our patient was also started on the same regimen. She completed her sixth cycle of chemotherapy 2 years back, as a part of her treatment plan. The multimodality treatment approach in our case might have improved the prognosis of synovial sarcomas. On her follow-up radiological scans, pulmonary nodules have decreased in size while left breast nodules are stable as of now.

As mentioned earlier, factors associated with a worse prognosis include tumour diameter exceeding 5 cm, incomplete surgical resection, local recurrence, patient age over 20 years, presence of the monophasic variant, and distant metastasis. Therefore, the extended survival observed in our patient is fortunately not the typical outcome despite the unfavourable prognosis.
